# Case of Meningitis in an Elephant

**Published:** 1881-01

**Authors:** Charles A. Todd

**Affiliations:** Medical Director Zoological Department; St. Louis


### CORRESPONDENCE.

### A CASE OF MENINGITIS IN THE ELEPHANT—POST-MORTEM.

EDITOR OF JOURNAL OF COMPARATIVE MEDICINE:—

I SEND you a history of the following case, which seems to be unique:

The African elephant at the St. Louis Zoological Garden (Department of the St. Louis Agricultural and Mechanical Association), not long after his removal into winter quarters, began to exhibit a capricious appetite which gave the keeper much anxiety, as in the course of a week or so he showed an indifference towards his grain feed (oats and Indian corn), as well as the bran; he finally refused grain, at the same time gradually falling away in flesh. Timothy and clover hay formed his chief diet during the last three weeks of his life. Corn, cooked and raw alike, was rejected; potatoes and turnips were at first eaten, but soon refused; apples were most acceptable, but, as may be supposed, he was not greatly indulged in that diet. The gradual but constantly increasing emaciation indicated serious disorder of some nature, though what it might be was not apparent. To improve the appetite, small doses of potassium iodide had been given for some three weeks before, and then discontinued, with expectation of readministration.

The evacuations were normal, the eye bright, the bodily movements natural, the condition of the skin as good as at any previous time. The only symptom, apart from the loss of appetite, that might be regarded as a token of sickness, was the occasional resting of his head against the wall; but it is said that, in the wild state, elephants sometimes sleep in that position. Another symptom was a peculiar spasmodic jerking of the abdomen that took place once in a while, without apparent cause. This, however, was slight, and only to be observed on close inspection; besides, it was a trick of old standing. The keeper thought he had become rather more vicious, showing a disposition to rush at him without provocation. Balls had been put upon his tusks last spring, to prevent damage to walls and fences. Still, there was nothing definite to suggest the actual outcome of his sickness, and neither the keeper nor myself suspected the brain to be the main seat of disorder. I prescribed an aloes purgative, combined with ginger and molasses, thinking that free evacuation of the bowels might improve the appetite, and relieve a probable dyspeptic condition. Human beings are apt to be not over good-natured when they have a poor digestion. The dose was such as is given the largest horse, and acted in twenty-four hours moderately, but moved the bowels sufficiently without causing diarrhœa. He seemed decidedly better; ate more freely and acted more naturally for several days, when, without any warning, he was seized at noon with a frenzy, broke loose from his fastenings, and charged about the carnivora-house, in which was his enclosure, with an unprecedented outbreak of anger, trumpeting and roaring, and raking the floor with his tusks. The assistant keeper fled for his life into a convenient closet, while the regular attendant manfully stuck by; but, expecting that the furious beast would break through the protecting railing, and set loose some of the carnivora that were already greatly excited, he opened the outer door, through which the elephant rushed into the open. In the park, he was temporarily held by winding a chain still attached to a hind leg about convenient trees, as he paused in the intervals of his madness. Towards evening he was coaxed, while partly quieted, into a log stable, and there fastened in. He then laid down, and about midnight died without more disturbance.

A post-mortem was made, with the aid of a number of assistants. The liver was the only abdominal organ that appeared diseased. This had undergone a fibrous degeneration (cirrhous?), much of the proper liver substance having disappeared. The stomach and bowels were filled with well masticated and digested food. The lungs were adherent to the chest walls, showing an old and extensive pleurisy. Heart normal. The chief disorder lay in the head. The membrane next the brain (the pia mater), on the upper surface of the brain, was much thickened, owing to a chronic inflammation, and one evidently of quite long standing, as the vessels passing into the pia resisted considerable traction. The brain substance, upon raising the pia, tore away with it. The vessels of the meninges were much congested, which state explained the sudden attack of madness. There was also some congestion of the lungs and liver, but this was a secondary matter.

It was fortunate that the elephant had been weakened by sickness, or in his frenzy much mischief might have been done. The animal was about two-thirds grown.

CHARLES A. TODD, M.D.,

*Medical Director Zoological Department.*

ST. LOUIS, Dec 17, 1880.

